# Development of the Incontinence Utility Index: estimating population-based utilities associated with urinary problems from the Incontinence Quality of Life Questionnaire and Neurogenic Module

**DOI:** 10.1186/s12955-014-0147-7

**Published:** 2014-10-08

**Authors:** Jesús Cuervo, Nacho Castejón, Kristin M Khalaf, Catherine Waweru, Denise Globe, Donald L Patrick

**Affiliations:** LASER Analytica, C/Azcárraga 12 A, 33010 Oviedo, Asturias Spain; Allergan Inc, Irvine, California USA; Department of Health Services, University of Washington, Seattle, Washington USA

**Keywords:** Overactive bladder, Urinary incontinence, Utility, Preference, Quality of life, Multi-attribute theory

## Abstract

**Background:**

Generic utility instruments may not fully capture the impact and consequences of urinary problems. Condition-specific preference-based measures, developed from previously validated disease-specific patient-reported outcomes instruments, may add relevant information for economic evaluations. The aim of this study was to develop a condition-specific preference-based measure, the Incontinence Utility Index (IUI), for valuing health states associated with urinary problems.

**Methods:**

A two-step process was implemented. First, an abbreviated health state classification system was developed from the Incontinence Quality of Life Questionnaire (I-QOL) and Neurogenic Module by applying Rasch modelling, classical psychometrical testing and expert criteria to data from two pivotal trials comprised of neurogenic detrusor overactivity (NDO) patients. Criterion, convergent validity and concordance with the original instrument was assessed in the abbreviated version. Then, a multi-attribute utility function (MAUF) was estimated from a representative sample of the UK non-institutionalized adult general population. Visual analogue and time-trade off (TTO) evaluations were applied in the elicitation process. Predictive validity of the MAUF was tested comparing estimated and direct utility scores.

**Results:**

The abbreviated health state classification system generated from the NDO sample contained 5 attributes with 3 levels of response and had adequate psychometrical properties: significant differences in scores according to the reduction in the frequency of urinary incontinence episodes [UIE] (p < 0.001); Spearman correlation coefficient with number of daily UIE = −0.43; p < 0.01 and Intraclass Correlation Coefficient (ICC, 95% CI) with the original version = 0.90 (0.89-0.91; p < 0.001). Next, 442 participants were interviewed (398 cases were valid, generating 2,388 TTO evaluations) to estimate the social preferences for derived health states. Mean age was 44.75 years (interquartile range 33.5-55.5) and 60.1% were female. An overall algorithm for the IUI was estimated and transformed onto a dead = 0.00 and full health = 1.00 scale. Model fits were acceptable (R-squared = 0.923 and 0.978). Predictive validity was adequate: ICC (95% CI) = 0.928 (0.648-0.985) and Mean of Absolute Differences = 0.038.

**Conclusions:**

The newly developed IUI is a preference-based measure for urinary problems related to NDO that provides general population-based utility scores with adequate predictive validity.

**Trial registration:**

ClinicalTrials.gov: NCT00461292, NCT00311376.

**Electronic supplementary material:**

The online version of this article (doi:10.1186/s12955-014-0147-7) contains supplementary material, which is available to authorized users.

## Background

Urinary problems, particularly when accompanied with urinary incontinence (UI), have been shown to significantly impact different domains of health-related quality of life (HRQoL) such as emotional well-being, performance of daily activities and social interaction [1], and have also been associated with economic burden [2,3] and lower productivity [1,4]. Neurogenic detrusor overactivity (NDO) is an etiology of UI that is caused by conditions such as multiple sclerosis (MS) or spinal cord injury (SCI). Detrusor overactivity is an involuntary bladder contraction during the filling phase of cystometry [5]. As a result of a disruption in the regulation of the micturition reflex, NDO patients frequently suffer from urinary symptoms including urgency and urinary urgency incontinence, which negatively affect their HRQoL [6,7].

A practical approach to evaluating the health states derived from a disease is through administration of existing generic preference-based instruments such as the EQ-5D [8,9], the Health Utility Index –Mark 2 or Mark 3- (HUI2 or HUI 3, respectively) [10,11] or the SF-6D [12,13]. These instruments are suitable across patient populations, regardless of the disease, allowing investigators to describe and compare important aspects of HRQoL and produce preference-based or utility scores. Although there is evidence to suggest that there is an underlying basic construct measured by the three generic instruments, it is well established that they produce different values and are not interchangeable [14-18]. Furthermore, there is controversy about their discriminative ability and sensitivity to detect clinically important changes in varying patient populations and consequently, these measures may not be the best choice for certain conditions [19], including urinary incontinence-related problems [20-23].

There are a number of condition-specific instruments available for patients with lower urinary tract symptoms and UI. Some commonly-used measures in clinical trials and outcomes research are the Overactive Bladder Questionnaire (OAB-q) [24], the King’s Health Questionnaire (KHQ) [25] and the Incontinence Quality of Life Questionnaire (I-QOL) [26-28]. These instruments have good psychometric properties in terms of reliability, construct and discriminant validity, and responsiveness [6,24,27,29]. Recently, new efforts have been focused on estimating utilities related to health states derived from these tools by means of surveying different samples of patients or general population from Europe and the US [30-32]. However, out of all these measures, only the I-QOL questionnaire includes a specific module for NDO patients developed from the needs-based model [26,27]. In addition, the validity of the I-QOL has been demonstrated in patients with neurogenic urinary incontinence [6]. Consequently, the overall aim of this research was to generate a preference-based measure from the I-QOL and its neurogenic set, the Incontinence Utility Index (IUI), by means of surveying a representative sample of the general population. This new instrument would represent a more comprehensive measure for valuing health states associated with urinary problems from a range of different etiologies.

## Methods

### Overview

#### The I-QOL Questionnaire and Neurogenic Module

The I-QOL is a self-administered disease-specific instrument comprised of 22 questions (5 point Likert scale) addressing three main domains: Avoidance and Limiting Behavior, Psychosocial Impact, and Social Embarrassment [26,27]. A global scale score is obtained by summing up the responses to all items and transforming the raw total score to a 0–100 scale (0-worst/100-best HRQoL). As noted before, the I-QOL was developed and validated among patients with stress UI and overactive bladder (OAB) [27] and has since been successfully tested on other patient populations, such as NDO patients [6] or patients with urgency UI who had not been adequately managed with anticholinergic therapy [28]. The additional module for patients with neurogenic bladder consists of 5 items about limiting caffeine drinks, worry about long-term effects of catheterization, accessibility and privacy in public toilets, bother associated with catheterizing, and bother associated with the use of pads or diapers.

A 2-stage process was used to develop the IUI from the I-QOL. The first stage was to use Rasch analysis and classical psychometrical tests to derive an abbreviated health state classification from the I-QOL and Neurogenic Module that is suitable for preference elicitation. The second stage was to conduct a preference elicitation survey to allow the estimation and validation of a multi-attribute utility function (MAUF) for the IUI.

### Deriving an abbreviated health state classification system from the I-QOL and the neurogenic module

Multi-Attribute Utility Theory (MAUT) is an approach that assigns utility weights to different outcomes by considering multiple attributes and the associated preferences reported by a given population, and then combining individual values into an overall utility measure. This process involves specifying a particular form for the utility function and the possible preference interactions among the attributes [10,11,33]. For MAUF estimation, it is important to include a range of aspects describing relevant consequences of a given disease on patients’ lives to ensure accuracy and sensitivity to change. The attributes should not be too large, however, so as not to increase respondents’ cognitive burden and make data collection impractical. The I-QOL, along with Neurogenic Module, generates a total of 5^27^ different health states. Hence, a psychometric analysis was required to extract a minimum but valid set of health states [34]. To this end, Rasch analysis and statistics from Classical Test Theory (CTT) were combined.

Rasch analysis is a scaling methodology that allows the examination of the hierarchical structure, unidimensionality and additivity of HRQoL measures [35]. Rasch methods may be used to identify and select items in an instrument that best cover the entire continuum of the underlying construct and remove redundant items [34,36,37]. Data dimensionality was investigated using the approach suggested by Linacre (1998) [38] and item responses were analysed using the Partial Credit Model [35], considering model fit, item and category locations, and differential item functioning (DIF) regarding sex, age group and etiology (i.e. MS or SCI). Model fit in the range 0.5-1.5 was considered acceptable, and items with centered locations and ordered categories as spread as possible were preferred. DIF was considered relevant when it was statistically significant and the difficulty difference between groups was over 0.5 logits. Additionally, an expert panel including the developer of the I-QOL and other experts in urology and psychometrics was convened to review the best items according to the results of the analyses. Finally, internal consistency (Cronbach’s α > 0.8), criterion validity (statistically significant differences in HRQoL according to the reduction in the frequency of UI episodes) and agreement with the original I-QOL (Intraclass correlation coefficient –ICC- ≥0.75) were tested to ensure that the abbreviated version met an acceptable standard in these properties. The sample used in this first stage has been described elsewhere [39,40]. Briefly, we pooled data from two randomized trials of onabotuliniumtoxinA (BOTOX®) 200U or 300U vs. placebo in adult patients with UI due to NDO. A total of 691 patients were enrolled in these two trials, 44.9% of whom had SCI and 55.1% of whom had MS. The primary time point was week 6; patients could request a second treatment after week 12 and were followed up to week 52.

### Weighting the health states defined by the new abbreviated health state classification system

#### Elicitation survey

A cross-sectional observational study was conducted between October and December 2012 to survey a representative sample (n = 442) of English-speaking, non-institutionalized adults from the general population in United Kingdom (UK). Participants were eligible to participate if they were willing to complete the interview process and to endorse their compliance with the quality standards of the survey. Written informed consent was obtained prior to participation. Exclusion criteria included cognitive impairment, suspicion of being under the effects of alcohol or narcotics use during the study visit, and any concurrent medical condition limiting their capacity to complete the evaluation.

Sample size was set to a minimum of 338 responders to enable the estimation of mean values with a confidence interval of ±0.032 points, and a standard deviation of 0.3 points, assuming a normal distribution of scores with a confidence interval of 95% (t-value = 1.96) [41]. However, given the complexity of the elicitation process and previous experiences [42], it was estimated that a maximum of 30% of the respondents would not be able to successfully complete all the proposed rating exercises. Hence, a total of 440 participants were interviewed.

Sampling was carried out in two steps: Cluster random sampling was first applied based on UK regions/postcodes. Subjects were then randomly selected from each region/postcode while monitoring other key socio-demographic variables (age, gender, education and employment status). The uniqueness of each participant’s identity was verified at recruitment and before the interview using their name and address. Single visits for interviews were face to face and conducted using a Computer Assisted Personal Interviewing methodology, at either a central location in major hubs across the UK, or by visiting respondents at home at an agreed time and date. Respondents were offered £20 for participating in the survey, plus an additional £5 for travel expenses if they were asked to come to a central location to participate.

A total of 10 professional interviewers with required qualifications and relevant experience in conducting face to face interviews collaborated in this research. They received intensive instruction, including role-playing sessions to ensure the quality of interviews. Interviewers were required to record the time needed to complete each interview immediately following each survey, and also to rate the degree of understanding and cooperation from participants along with the overall quality of the interview.

Opinion Health^©^ was in charge of data collection which was conducted according to the Code of Conduct of the Market Research Society [43], European Pharmaceutical Market Research Association [44] and qualitative recruitment best practice outlined by The Association of Qualitative Research [45].

All procedures and materials were tested in a pilot study (n = 13) to identify any practical problems with data collection and to validate instruments and materials used prior to the study. Following the pilot study, additional debriefing sessions were conducted with the interviewers in order to ensure that interviews would be conducted in a systematic way to minimize possible sources of bias.

### Modelling space of the new abbreviated health state classification system

All health states were carefully chosen to allow estimation of the 5 single-attribute utility functions, each of the attribute weights in the multi-attribute utility function, and the interaction term (see *MAUF estimation* below). A total of 16 different states were evaluated (5 single-attributes and 11 multi-attributes).

Respondents were asked to assume a time horizon range of 30 years to better reflect the chronic nature of the health states presented. This period of time is close to the average expected years of life for a middle-age person according to UK life expectancy tables. The time horizon and the chronicity of states were discussed with all the participants before proceeding with the interview.

**Figure 1:**
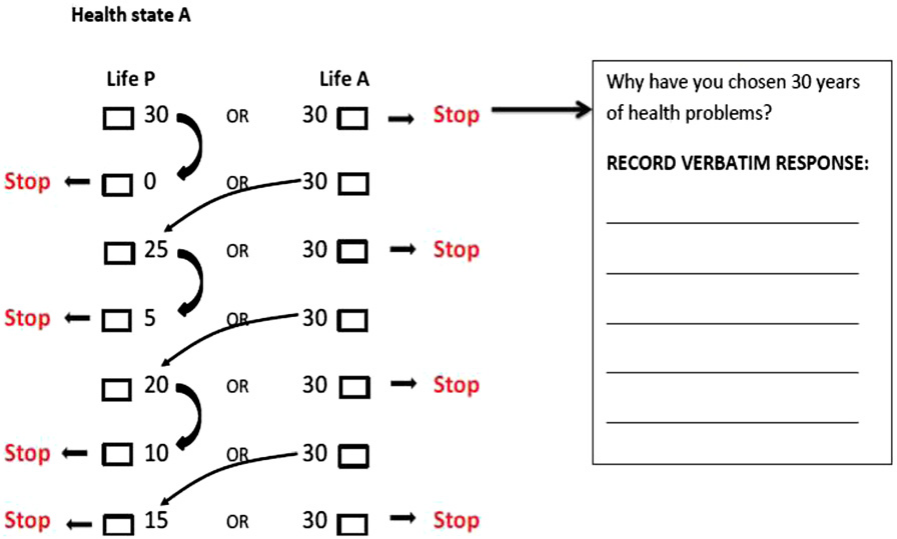
**Presentation diagram of the time trade-** **off technique.** Life P= The most desirable health state/Full health/The best health state imaginable. Life A= A given health stated derived from the abbreviated health state classification system.

In addition to basic socio-demographic variables, each participant responded to the following evaluation exercises required for MAUF estimation:

- Visual Analogue Scale (VAS) rating of single-attribute utility functions. For each attribute (n = 5), VAS rating of the intermediate level was conducted on a thermometer feeling scale anchored at 0- the least desirable or the worst level at each attribute and 100- the most desirable or best level at each attribute.
- VAS rating of multi-attribute health states: A total of 5 corner states, 3 intermediate or marker states and 3 anchoring states were performed. Intermediate or marker states were chosen to ensure the evaluation of a wide range of levels within the 5 targeted attributes, enhancing the precision of estimations. Anchoring references were: 0- the least desirable health state defined by the attributes (health state W) or dead - and 100-the most desirable health state or perfect health, defined as the conjunction of the top level at each attribute (health state P) [11]. It is important to note the lowest anchor states were chosen depending on each participant’s preferences. Therefore, for those respondents who declared that being dead was preferable to health state W, being dead was measured on a scale ranging from 0-health state W to 1- the most desirable/perfect health scale. In contrast, for those respondents who valued being dead as worse than health state W, health state W was then valued on a scale ranging from 0-dead to 1-the most desirable/perfect health scale.
- Time Trade-Off rating (TTO) for power function estimation and for evaluation of MAUF predictive validity. A total of 6 different states were assessed: 3 corner states and all the intermediate or marker states previously described. During the elicitation process, a “*ping-pong*” presentation was used to converge on an indifference point between the alternatives (Figure 1). All participants were requested to think about the differences between the health states compared in each exercise, always keeping in mind that all other important, broader factors would remain constant (family, job, friends, income, etc.) under all the presented scenarios.

### MAUF estimation

A person-mean utility approach was used to estimate a general utility function based on community responses [10,11,42]. MAUF forms include the additive form, the multiplicative form and the multi-linear form [10]. A detailed introduction to the principles for MAUF estimation can be found in the literature [10,11,33]. Considering the reduced version of the I-QOL comprises 5 health domains, the multiplicative MAUF is reasonable and has empirical support [10,33]. Since it is easier for respondents to imagine corner states (with one attribute at its worst level and the rest of attributes at their best level), this function is normally expressed in terms of disutility (*ū)* which is just the complement of the utility:

1 $$ \overline{u}=\left(\frac{1}{c}\right)\left[{\displaystyle \prod_{j=1}^5\left(1+c{c}_j{\overline{u}}_j\right)-1}\right] $$

where

2 $$ 1+c={\displaystyle \prod_{j=1}^5\left(1+c{c}_j\right)} $$

*c*_j_ is equal to the disutility of the corner state for attribute j and represents the weight attached to that attribute. If the sum of all *c*_*j*_ equals 1 then the additive model holds. c is the interaction term and results from solving Equation 2. The methods applied in this research are similar to those followed to develop the HUI-3 [11]. A complete description of the statistical approach can be found in the Additional file 1. Briefly, after confirming the consistency of respondents’ ratings, the sample was split in 2 groups according to the health state they considered less preferable (dead or the worst health state possible in the abbreviated health state classification system). Hence, two separate power functions [46] were calculated to convert VAS values (v) into utilities (u) and the adjusted overall person mean scores were calculated with utility values ranging from Dead = 0.00/P = 1.00 scale. Next, the relative weight of each parameter (c_j_ in disutility terms or w_j_ in utility terms) and its interaction form were studied for each group and for the overall sample and finally, the IUI algorithm was estimated.

### Predictive validity of the IUI

The accuracy of the algorithm was analysed by comparing estimated and directly measured utilities (TTO) on intermediate states. A number of statistics were computed:

- Sum of total differences (Σ differences): Σ differences = Σ (predicted *u*_*j*_ – observed Person-Mean *u*_*j*_)
- Mean of differences (MD): MD = [Σ (predicted *u*_*j*_ – observed Person-Mean *u*_*j*_)/n_j_]
- Mean of absolute differences (MAD): MAD = [Σ |(predicted *u*_*j*_ – observed Person-Mean *u*_*j*_)|/n_j_]
- Overall standard deviation (OSD) of differences: OSD = [(Σ (predicted *u*_*j*_ – observed Person-Mean *u*_*j*_)^2^)/(n_j_-1)]
- ICC between estimated and directly measured scores [47].

For these metrics, values as close to zero as possible are preferred, except for the ICC, interpreted as any correlation. The statistical packages WinSteps software version 3.72.3 and Stata10 along with the spreadsheet Excel 2007 (Microsoft) were used for the analyses presented in this manuscript [48,49].

## Results

### I-QOL reduction

Outputs from Rasch analysis are presented in Table 1. No age related DIF was identified. Items 1, 3, 4 and 10 in the I-QOL and 2 and 4 in the Neurogenic Module had etiology related DIF. Item 10 from the I-QOL and 2 and 4 from the Neurogenic Module had sex related DIF. These six items were removed from the selection based exclusively on the results of the Rasch analysis. Additionally, as previously stated, an expert panel then proceeded to consider the results of the analysis jointly with the item content, to reach the final selection of 5 items considered to represent a set of complementary attributes. The 5 response categories were collapsed into 3 to simplify health state valuation, yielding the abbreviated health state classification system (Table 2). This final version proved to be internally consistent and valid for NDO patients according to the psychometric analyses presented in Table 3: at week 6, the abbreviated health state classification proved to have adequate ability to detect changes in those patients who showed a reduction in incontinence episodes (responders), and the association between daily incontinence episodes and health state classification scores was considered adequate. Furthermore, the level of agreement between the original I-QOL and the abbreviated health classification system was high (ICC = 0.90; 95% CI: 0.89-0.91) and statistically significant (p < 0.001).

**Table 1:** **Summary of Rasch outputs**

**Item**	**Measure**	**Infit MeanSq**	**Outfit MeanSq**	**Pt. measure correlation**	Etiology DIF*		**Sex DIF**	
					**Contrast**	**p**	**Contrast**	**p**
I-QOL01	0.17	1.04	1.12	0.48	*0.60*	*0.000*	*−0.47*	*0.000*
I-QOL02	−1.07	1.25	1.33	0.50	0.23	0.002	−0.29	0.000
I-QOL03	−0.85	1.23	1.32	0.51	*0.59*	*0.000*	−0.41	0.000
I-QOL04	0.26	1.05	1.19	0.47	*0.60*	*0.000*	−0.40	0.000
I-QOL05	−0.29	0.97	1.01	0.55	−0.25	0.001	0.10	0.209
I-QOL06	−0.15	0.88	0.83	0.59	0.00	1.000	0.14	0.060
I-QOL07	0.22	0.74	0.70	0.61	−0.04	0.595	0.00	1.000
I-QOL08	−0.32	1.09	1.12	0.51	−0.35	0.000	0.27	0.000
I-QOL09	0.51	0.75	0.74	0.60	0.16	0.056	−0.05	0.557
I-QOL10	0.22	0.94	0.97	0.52	*0.76*	*0.000*	*−0.53*	*0.000*
I-QOL11	0.25	0.86	0.83	0.56	−0.21	0.013	0.19	0.029
I-QOL12	0.27	1.01	1.10	0.49	0.18	0.025	−0.09	0.230
I-QOL13	−0.12	1.10	1.12	0.49	0.33	0.000	−0.19	0.010
I-QOL14	0.44	0.72	0.68	0.61	0.00	1.000	−0.03	0.761
I-QOL15	−0.29	0.92	0.92	0.58	0.12	0.103	0.00	1.000
I-QOL16	−0.46	0.82	0.81	0.62	−0.06	0.432	0.00	1.000
I-QOL17	−0.12	0.75	0.72	0.63	0.00	1.000	0.00	1.000
I-QOL18	0.76	0.75	0.69	0.57	−0.22	0.017	0.00	1.000
I-QOL19	0.91	0.95	0.82	0.48	−0.20	0.054	0.13	0.195
I-QOL20	0.46	0.94	0.92	0.51	−0.06	0.521	−0.08	0.376
I-QOL21	−0.45	0.98	0.96	0.57	0.13	0.075	−0.11	0.132
I-QOL22	−0.47	1.24	1.37	0.48	−0.43	0.000	0.29	0.000
Neurog1†	−0.26	1.20	1.29	0.47	0.00	1.000	0.00	1.000
Neurog2	−0.23	1.33	1.61	0.42	*−0.61*	*0.000*	*0.57*	*0.000*
Neurog3	0.95	1.06	0.99	0.42	−0.24	0.025	0.18	0.099
Neurog4	−0.51	1.56	1.85	0.39	*−0.57*	*0.000*	*0.51*	*0.000*
Neurog5	0.15	1.14	1.16	0.46	−0.11	0.128	−0.12	0.096

*Differential Item Functioning; †Neurogenic Module of the I-QOL.

**Table 2:** **The abbreviated health state classification system derived from the I**-**QOL and Neurogenic Module**

**Attributes**	**Levels of response**	Score*
I-QOL Item5: Depression	I feel not at all depressed because of my urinary problems or incontinence	5
	I feel somewhat depressed because of my urinary problems or incontinence	3
	I feel extremely depressed because of my urinary problems or incontinence	1
I-QOL Item8: Urine Smell	I do not worry at all about other people smelling urine on me	5
	I worry somewhat about other people smelling urine on me	3
	I worry a very great deal about other people smelling urine on me	1
I-QOL Item13: Sleep	I have no difficulty getting a good night’s sleep because of my urinary problems or incontinence	5
	I have some difficulty getting a good night’s sleep because of my urinary problems or incontinence	3
	I have extreme difficulty getting a good night’s sleep because of my urinary problems or incontinence	1
I-QOL Item19: Bladder Control	I feel I have control over my bladder	5
	I feel I have some control over my bladder	3
	I feel I have no control over my bladder	1
I-QOL Item20: Drinks	I have to be not at all careful about what or how much I drink because of my urinary problems or incontinence	5
	I have to be somewhat careful about what or how much I drink because of my urinary problems or incontinence	3
	I have to be extremely careful about what or how much I drink because of my urinary problems or incontinence	1

*Summary raw score was transformed into a 0 (worst health status) – 100 (best health status) scale with the following algorithm: Scale score of the abbreviated form = (the sum of the 5 items - lowest possible score)* 100/possible raw score range.

**Table 3:** **Psychometric properties of the abbreviated health state classification system in neurogenic detrusor overactivity patients**

Psychometric properties*						
**Criterion validity: differences in the scale score of the abbreviated health state classification system (0: worst health status – 100: best health status) according to the reduction in the frequency of urinary incontinence episodes at week 6)**						
		**N**	**Mean**	**95% CI Lower L.**	**95% CI Upper L.**	**p**
Scale score at Day 1	Reduction <50%	225	35.11	32.73	37.49	0.242
	50% < = Reduction <100%	224	35.04	32.75	37.34	
	100% Reduction	188	37.93	35.40	40.45	
Scale score at Week 6	Reduction <50%	222	38.69	36.14	41.25	<0.001
	50% < = Reduction <100%	224	54.33	54.62	57.04	
	100% Reduction	186	70.81	67.72	73.89	
**Convergent validity Spearman correlation coefficient**						
		**Daily Incontinence episodes**				
Scale score at Day 1		−0.22				<0.01
Scale score at Week 6		−0.43				

*Overall internal consistency of the abbreviated health state classification system was adequate (Cronbach’s α = 0.814). Regarding construct validity, Principal Component analysis highlighted one principal component (57.5% of explained variance. All the items were highly correlated with that component r_xy_ ≥ 0.73).

### Weighting the health states derived from the I-QOL

Complete descriptions of the multi-attribute health states and the sample are presented in Tables 4 and 5. A total of 442 interviews were completed, however, 44 cases were withdrawn because they presented at least one inconsistency in their ratings: if VAS values for a given corner state (health states A to E, Table 4) were lower than the VAS values of comparable marker states (M1 to M3, Table 4), n = 50 (please note some participants provided more than one inconsistent answer); or if the value of any corner or marker states were lower than the VAS value of the least desirable health state, n = 24. Only those participants successfully completing all the rating exercises were included, n = 398, generating a total of 2,388 TTO evaluations. With respect to interview quality, 97.7% were performed with full cooperation of the respondent, 84.7% of participants thought carefully before answering, and 84.9% experienced very little or no problems completing the survey. Moreover, mean time required to complete the survey was 30.2 minutes (Standard deviation -SD- 10.9) and the vast majority of interviewers rated the quality as good or very good (94.2%) with less than 1% of interviews being considered of inferior quality.

**Table 4:** **Multi**-**attribute health states used in preferences elicitation**

Health states*	**Depression**	**Urine smell**	**Sleep**	**Bladder control**	**Drinks**
A^†^	3	1	1	1	1
B	1	3	1	1	1
C^†^	1	1	3	1	1
D^†^	1	1	1	3	1
E	1	1	1	1	3
M1^†^	1	2	3	1	2
M2^†^	3	1	1	2	2
M3^†^	1	2	1	3	3
P	1	1	1	1	1
W	3	3	3	3	3
Dead	--	--	--	--	--

^*^Levels of all attributes were coded as follows: 1-best possible level, 2-intermediate level and 3-worst possible level. All health states were rated with a visual analogue scale (0–100). A, B, C, D, E: corner states. M1, M2, M3: intermediate/marker states. *P*: Best health state possible. *W*: Worst health state possible.

^†^Health states also evaluated with a time trade-off technique.

**Table 5:** **Description of participants in the elicitation survey (valid cases, n= 398)**

**Variable**		**n**	**%**
Gender	Female	239	60.1
Region	North East	20	5.0
	North West	38	9.5
	Yorkshire and The Humber	35	8.8
	Midlands	55	13.8
	East of England	32	8.0
	London	47	11.8
	South East	68	17.1
	South West	37	9.3
	Scotland	36	9.0
	Northern Ireland	10	2.5
	Wales	20	5.0
Education	Some secondary school	14	3.5
	GCSE or equivalent	71	17.8
	‘A’ level or equivalent	71	17.8
	Diploma or certificate of higher education	66	16.6
	Bachelor’s degree or equivalent	107	26.9
	Master’s or Doctoral degree/Post graduate certificate	69	17.3
Employment status	Working full-time	150	37.7
	Working part-time	56	14.1
	Not working	55	13.8
	Looking for work	4	1.0
	Student	36	9.0
	Retired	65	16.3
	Self employed	32	8.0
Age	Mean (SD)	44.75	14.6
Groups of Age	18-29	73	18.3
	30-39	92	23.1
	40-49	90	22.6
	50-59	73	18.3
	60-69	54	13.6
	>70 years old	16	4.0
Suffering a Chronic Disease	Yes	126	31.7
Acute disease	Yes	35	8.8
Bladder symptoms	Yes	145	36.4
Bladder symptoms in family or friends	Yes	191	48.0

A majority of respondents were female (60.1%), mean age was 44.75 years (SD14.6); 60.8% had at least a diploma education (2 years of college) and a similar percentage were employed (59.8%). With respect to their health status, 31.7% reported a chronic illness and 8.8% an acute disease. Regarding previous experience, 36.4% declared they had suffered symptoms associated with OAB or UUI and 48.0% recognized some of these problems in their relatives or friends.

With respect to participants’ preferences about the worst state described by the abbreviated health state classification system and dead, most of them (n = 294, 73.9%) stated they would prefer living the next 30 years in health state W (Group B), while the rest (n = 104, 26.1%) preferred being dead to living in health state W (Group A).

### MAUF estimation and final algorithm of the Incontinence Utility Index

Trimmed values (10%) were fitted separately for each group based on power functions (Equation 3 in Additional file 1) and natural log transformations (Equation 4 in Additional file 1) to convert mean VAS (v) into utility scores (u). Regression models yielded good fit (R^2^ group A = 0.923 and R^2^ group B = 0.978) and power functions resulted as follows: Group A, u = 1-(1-v)^1.229^ and Group B, u = 1-(1-v)^0.841^. Estimates of the relative weight of each attribute fitted in the perfect health = 0 and worst state = 1 for Group A were: c1 = 0.393, c2 = 0.450, c3 = 0.387, c4 = 0.562 and c5 = 0.283 (Σcj = 2.076; c = −0.911). For Group B: c1 = 0.636, c2 = 0.640, c3 = 0.616, c4 = 0.775 and c5 = 0.490 (Σcj = 3.158; c = −0.994). From these results it was seen that the multiplicative form was an appropriate form.

Final utilities were calculated based on the prevalence proportion in Person-Mean A and Person-Mean B groups (both in W = 0.00/P = 1.00 scale): uj = (104* Person-Mean A uj + 294 * Person-Mean B uj -re-scaled-)/398. A positive linear transformation was applied to re-scale the utilities into a dead = 0.00 / P = 1.00 scale to facilitate comparisons with other utility measures. Table 6 shows utility weights estimated for the multi-attribute health states defined in Table 4. The disutility weights estimated for each attribute from the overall sample were: c1 = 0.470, c2 = 0.484, c3 = 0.456, c4 = 0.590 and c5 = 0.358 (Σcj = 2.357; c = −0.951). Once again the results rejected the linear additive form and showed that all attributes were preference complements. The five single attribute utility coefficients and the overall MAUF are presented in Table 7 with possible scores ranging from 0.036 (worst health state) to 1 (perfect health).

**Table 6:** **Estimated overall utility scores**

**Health state**	**n**	***u*** *	***u’*** ^***†***^
A	398	0.427	0.530
B	398	0.409	0.516
C	398	0.444	0.544
D	398	0.280	0.410
E	398	0.564	0.642
M1	398	0.259	0.392
M2	398	0.243	0.379
M3	398	0.185	0.331
P	398	1	1
D	398	−0.219	0
W	398	−0.037	0.150

*u = (104* Person-Mean A uj + 294. *Person-Mean B uj -re-scaled-)/398.

^†^Positive linearly transformed into a dead = 0.00/P = 1.00 scale.

**Table 7:** **Single and Multi**-**attribute utilities**

**Single-** **attribute utility scores**					
**Level**	**Depression**	**Urine Smell**	**Sleep**	**Bladder Control**	**Drinks**
1	1.00	1.00	1.00	1.00	1.00
2	0.600	0.457	0.613	0.627	0.655
3	0.178	−0.034	0.178	0.178	0.178
**Final Multi-attribute utility function coefficients (p = 0.051)**					
**Level**	**Depression (w1)**	**Urine Smell (w2)**	**Sleep (w3)**	**Bladder Control (w4)**	**Drinks (w5)**
1	1.00	1.00	1.00	1.00	1.00
2	0.821	0.750	0.832	0.791	0.883
3	0.633	0.524	0.644	0.539	0.721
Final algorithm: u* = 1.051 (w1 * w2 * w3 * w4 * w5) – 0.051					

*u is the utility of a health state (number of possible health states = 243). Dead = 0.0 and Perfect Health = 1.0.

### Predictive validity of the MAUF

Mean utility scores of marker states directly elicited on the TTO were compared against those estimated by the MAUF to test its predictive validity. The results were as follows: Σ differences = −0.038; MD = −0.013; MAD = 0.038; OSD = 0.004 and ICC (95% CI) = 0.928 (0.648-0.985). Thus, the calculated MAUF showed a very slight tendency to underpredict directly elicited utilities. Moreover, the level of agreement found between both methods (ICC) was good and only 7.2% of variability could not be attributed to subjects.

## Discussion

In this study, a new utility index, the IUI, has been estimated from the abbreviated health state classification system derived from I-QOL and its neurogenic module by means of eliciting preferences from a representative sample of UK adult general population [50]. The abbreviated I-QOL version was internally consistent and able to capture clinically important differences in clinical status of NDO patients with UI (i.e. changes in HRQoL according to reductions in the average number of IU episodes per week). Furthermore, a high level of agreement was found between the reduced version and the original I-QOL, confirming the appropriateness of the abbreviated health state classification system of 5 domains and its modelling space for utility estimation. Moreover, all the psychometric procedures undertaken to reduce the I-QOL have been successfully applied previously [30,36,51] and have been recently recommended [34].

Regarding the elicitation process, methods applied are consistent with those used to develop one of the most widespread and robust generic utility measures, the HUI [10,11]. As has occurred in previous publications, the additive model was rejected in this study [10,11,42] and attributes were preference complements: for instance, the perceived limitation associated with being depressed and not having bladder control is greater than the separate effect of being depressed and not having bladder control, but smaller than the sum of these two problems.

In addition, predictive validity of IUI scoring algorithm was confirmed after comparing the direct utility values and those estimated for the final algorithm. Recognizing that IUI algorithm showed a slight tendency to underpredict the directly elicited utilities, error size was small and comparable to those errors reported for other utility instruments [11]. What is more, the ICC between direct and indirect values showed an adequate level of agreement.

Generic preference-based indices have historically been the most commonly used means of estimating utilities across a variety of conditions. However, substantial research has been conducted which shows the limitations of these instruments in different conditions [19-22], as well as the lack of concordance between the utility values obtained from their application [14-16,18,52,53]. As a result, the development of condition-specific preference-based measures has been gaining ground in recent years [30-32,54].

There are published studies focused on obtaining utility scores from condition-specific instruments for urinary problems. An algorithm has been generated to derive utilities from the KHQ by eliciting preferences from a sample of UI patients [31]. Kay et al. (2013) [32] mapped EQ-5D utility scores from the I-QOL among patients with neurogenic and idiopathic OAB using cross-sectional data from Europe and the US. Finally, Yang et al. estimated a population’s preference-based index from the OAB-q, the OAB-5D [30]. Consequently, although the IUI was derived from the I-QOL and its specific module for neurogenic patients, the OAB-5D is the most similar instrument because its modelling space was also obtained from applying Rasch, preference elicitation involved TTO evaluations, and also incorporates general population preferences. Nevertheless, relevant differences lay in the characteristics of the samples used in the reduction process (we specifically used NDO patients) and in the estimation models applied to derive the utility scores since OAB-5D followed the methods described previously for the SF-6D [12] and we computed a MAUF in accordance with the HUI latest versions [10,11]. Despite these differences, mean absolute error/differences in both measures are comparable (OAB-5D: 0.044 versus IUI: 0.038). Hence, additional research is needed to compare performance of each respective measure in the same populations (i.e. criterion validity, responsiveness and influence on cost-effectiveness ratios).

Despite the fact that the MAUF has proven robust, there are a number of limitations in this research. It should be noted that we used TTO evaluations instead of the Standard Gamble (SG). Although SG is considered the preferred technique to collect subjects’ preferences, TTO is a legitimate and extensively used technique, generally considered easier to understand and less time consuming [30,55]. Preferences were elicited from a UK-specific population, so caution should be used before applying the algorithm to other countries, especially if the population is expected to perceive urinary problems differently. Additionally, as a condition-specific preference-based measure, the IUI may suffer from some potential risks in terms of comparability of results [34]. The risk of *focusing effects* (i.e. cognitive bias that occurs when participants place too much importance on the problems associated with the health states presented to them compared with other conditions) was obviated as best as possible by clearly stating throughout the preference elicitation process that, apart from the health states described by the reduced version of the I-QOL, other important aspects of life (i.e. family, economic situation, friends, job, etc.) would remain constant.

Another source of limitations referred to as *anchoring* (defining a specific upper anchor that could make comparability across other preference-based instruments problematic) was also anticipated. Consequently, the upper limits during the evaluation process were defined as the most desirable health state, the best health state imaginable, or full health to best facilitate comparisons with other scales. Finally, while the 30-year time horizon was set to illustrate the chronicity of health states, this time frame may not have been the most appropriate for participants under 30 years of age (18.3%) or, particularly for those older than 60 years (17.6%). Thus, this time horizon may result in some over/underestimations during TTO exercises with these subsamples [56,57].

## Conclusions

The I-QOL and the IUI are valid-in-population measures for measuring HRQoL and utilities, respectively, associated with urinary problems. Although the IUI is the first utility measure that has been developed for a specific subset of patients with urinary symptoms (NDO population), it is important to note that the final selection of attributes included in the IUI is from the original I-QOL, with no items utilized from the Neurogenic Module. Hence, investigators may test its applicability in other relevant subsamples. It is worth noting that the use of a representative sample of general population to value its health states may ease the application of this instrument in new subsets of patients suffering from urinary problems. New research is currently underway to confirm the soundness of the IUI modelling space on idiopathic OAB patients and to study the responsiveness and the minimally important differences of the IUI in both NDO and idiopathic OAB populations. These insights will be of value to future researchers using the IUI instrument which is intended to complement utility estimates provided by generic instruments to support decision-making with reliable, valid and understandable information presented on a similar scale.

## Additional file

Additional file 1
**Estimating a multi-attribute utility function (MAUF) for the abbreviated health state classification resulted from the I-QOL and its neurogenic module.**

## Abbreviations

BOTOX: OnabotulinumtoxinA
CTT: Classical test theory
DIF: Differential item functioning
EQ-5D: EuroQol-5 dimension
HRQOL: Health-related quality of life
HUI: Health utility index
ICC: Intraclass correlation coefficient
I-QOL: Incontinence quality of life questionnaire
IUI: Incontinence utility index
KHQ: King’s health questionnaire
MAD: Mean of absolute differences
MAUF: Multi-attribute utility function
MD: Mean of differences
MS: Multiple sclerosis
NDO: Neurogenic detrusor overactivity
OAB: Overactive bladder
OAB-5D: Overactive bladder-5 dimensional
OAB-q: Overactive bladder questionnaire
OSD: Overall standard deviation of differences
PCA: Principal component analysis
SCI: Spinal cord injury
SF-6D: Short form-6 dimension
TTO: Time-trade off
UI: Urinary incontinence
UK: United Kingdom
VAS: Visual analogue scale

## References

[1] Coyne KS, Sexton CC, Kopp ZS, Ebel-Bitoun C, Milsom I, Chapple C (2011). The impact of overactive bladder on mental health, work productivity and health-related quality of life in the UK and Sweden: results from EpiLUTS. BJU Int.

[2] Ganz ML, Smalarz AM, Krupski TL, Anger JT, Hu JC, Wittrup-Jensen KU, Pashos CL (2010). Economic costs of overactive bladder in the United States. Urology.

[3] Reeves P, Irwin D, Kelleher C, Milsom I, Kopp Z, Calvert N, Lloyd A (2006). The current and future burden and cost of overactive bladder in five European countries. Eur Urol.

[4] Wu EQ, Birnbaum H, Marynchenko M, Mareva M, Williamson T, Mallett D (2005). Employees with overactive bladder: work loss burden. J Occup Environ Med.

[5] Abrams P, Artibani W, Cardozo L, Dmochowski R, van Kerrebroeck P, Sand P: **Reviewing the ICS 2002 terminology report: the ongoing debate.** *Neurourol Urodyn* 2009, **28:**287. 10.1002/nau.20737 19350662

[6] Schurch B, Denys P, Kozma CM, Reese PR, Slaton T, Barron R (2007). Reliability and validity of the Incontinence Quality of Life questionnaire in patients with neurogenic urinary incontinence. Arch Phys Med Rehabil.

[7] Tapia CI, Khalaf K, Berenson K, Globe D, Chancellor M, Carr LK: **Health-related quality of life and economic impact of urinary incontinence due to detrusor overactivity associated with a neurologic condition: a systematic review.** *Health Qual Life Outcomes* 2013, **11:**13. doi:10.1186/1477-7525-11-13. 13–11. 10.1186/1477-7525-11-13 PMC3606444 23369111

[8] The EuroQol Group (1990). EuroQol–a new facility for the measurement of health-related quality of life. Health Policy.

[9] Dolan P (1997). Modeling valuations for EuroQol health states. Med Care.

[10] Torrance GW, Feeny DH, Furlong WJ, Barr RD, Zhang Y, Wang Q (1996). Multiattribute utility function for a comprehensive health status classification system. Health Utilities Index Mark 2. Med Care.

[11] Feeny D, Furlong W, Torrance GW, Goldsmith CH, Zhu Z, DePauw S, Denton M, Boyle M (2002). Multiattribute and single-attribute utility functions for the health utilities index mark 3 system. Med Care.

[12] Brazier J, Roberts J, Deverill M (2002). The estimation of a preference-based measure of health from the SF-36. J Health Econ.

[13] Brazier J, Usherwood T, Harper R, Thomas K (1998). Deriving a preference-based single index from the UK SF-36 Health Survey. J Clin Epidemiol.

[14] Franks P, Hanmer J, Fryback DG (2006). Relative disutilities of 47 risk factors and conditions assessed with seven preference-based health status measures in a national U.S. sample: toward consistency in cost-effectiveness analyses. Med Care.

[15] O’Brien BJ, Spath M, Blackhouse G, Severens JL, Dorian P, Brazier J (2003). A view from the bridge: agreement between the SF-6D utility algorithm and the Health Utilities Index. Health Econ.

[16] Conner-Spady B, Suarez-Almazor ME (2003). Variation in the estimation of quality-adjusted life-years by different preference-based instruments. Med Care.

[17] Pinto AM, Kuppermann M, Nakagawa S, Vittinghoff E, Wing RR, Kusek JW, Herman WH, Subak LL (2011). Comparison and correlates of three preference-based health-related quality-of-life measures among overweight and obese women with urinary incontinence. Qual Life Res.

[18] McDonough CM, Tosteson AN (2007). Measuring preferences for cost-utility analysis: how choice of method may influence decision-making. Pharmacoeconomics.

[19] Papaioannou D, Brazier J, Parry G (2011). How valid and responsive are generic health status measures, such as EQ-5D and SF-36, in schizophrenia? a systematic review. Value Health.

[20] Haywood KL, Garratt AM, Lall R, Smith JF, Lamb SE (2008). EuroQol EQ-5D and condition-specific measures of health outcome in women with urinary incontinence: reliability, validity and responsiveness. Qual Life Res.

[21] Oh SJ, Ku JH (2006). Is a generic quality of life instrument helpful for evaluating women with urinary incontinence?. Qual Life Res.

[22] Finkelstein MM, Skelly J, Kaczorowski J, Swanson G: **Incontinence Quality of Life Instrument in a survey of primary care physicians.** *J Fam Pract* 2002, **51:**952. 12485550

[23] Davis S, Wailoo A: **A review of the psychometric performance of the EQ-5D in people with urinary incontinence.** *Health Qual Life Outcomes* 2013, **11:**20. 10.1186/1477-7525-11-20 PMC3622573 23418844

[24] Coyne K, Revicki D, Hunt T, Corey R, Stewart W, Bentkover J, Kurth H, Abrams P (2002). Psychometric validation of an overactive bladder symptom and health-related quality of life questionnaire: the OAB-q. Qual Life Res.

[25] Kelleher CJ, Cardozo LD, Khullar V, Salvatore S (1997). A new questionnaire to assess the quality of life of urinary incontinent women. Br J Obstet Gynaecol.

[26] Wagner TH, Patrick DL, Bavendam TG, Martin ML, Buesching DP (1996). Quality of life of persons with urinary incontinence: development of a new measure. Urology.

[27] Patrick DL, Martin ML, Bushnell DM, Yalcin I, Wagner TH, Buesching DP (1999). Quality of life of women with urinary incontinence: further development of the incontinence quality of life instrument (I-QOL). Urology.

[28] Patrick DL, Khalaf KM, Dmochowski R, Kowalski JW, Globe DR (2013). Psychometric performance of the incontinence quality-of-life questionnaire among patients with overactive bladder and urinary incontinence. Clin Ther.

[29] Reese PR, Pleil AM, Okano GJ, Kelleher CJ (2003). Multinational study of reliability and validity of the King’s Health Questionnaire in patients with overactive bladder. Qual Life Res.

[30] Yang Y, Brazier J, Tsuchiya A, Coyne K (2009). Estimating a preference-based single index from the Overactive Bladder Questionnaire. Value Health.

[31] Brazier J, Czoski-Murray C, Roberts J, Brown M, Symonds T, Kelleher C (2008). Estimation of a preference-based index from a condition-specific measure: the King’s Health Questionnaire. Med Decis Making.

[32] Kay S, Tolley K, Colayco D, Khalaf K, Anderson P, Globe D (2013). Mapping EQ-5D Utility Scores from the Incontinence Quality of Life Questionnaire among Patients with Neurogenic and Idiopathic Overactive Bladder. Value Health.

[33] Torrance GW, Furlong W, Feeny D, Boyle M (1995). Multi-attribute preference functions. health utilities index. Pharmacoeconomics.

[34] Brazier J, Rowen D, Mavranezouli I, Tsuchiya A, Young T, Yang Y, Barkham M, Ibbotson R (2012). Developing and testing methods for deriving preference-based measures of health from condition-specific measures (and other patient-based measures of outcome). Health Technol Assess.

[35] Wright B, Masters G (1982). Rating Scale Analysis. Rasch Measurement.

[36] Young T, Yang Y, Brazier JE, Tsuchiya A, Coyne K (2009). The first stage of developing preference-based measures: constructing a health-state classification using Rasch analysis. Qual Life Res.

[37] Mulhern B, Smith SC, Rowen D, Brazier JE, Knapp M, Lamping DL, Loftus V, Young TA, Howard RJ, Banerjee S (2012). Improving the measurement of QALYs in dementia: developing patient- and carer-reported health state classification systems using Rasch analysis. Value Health.

[38] Linacre JM (1998). Detecting multidimensionality: which residual data-type works best?. J Outcome Meas.

[39] Cruz F, Herschorn S, Aliotta P, Brin M, Thompson C, Lam W, Daniell G, Heesakkers J, Haag-Molkenteller C (2011). Efficacy and safety of onabotulinumtoxinA in patients with urinary incontinence due to neurogenic detrusor overactivity: a randomised, double-blind, placebo-controlled trial. Eur Urol.

[40] Ginsberg D, Gousse A, Keppenne V, Sievert KD, Thompson C, Lam W, Brin MF, Jenkins B, Haag-Molkenteller C (2012). Phase 3 efficacy and tolerability study of onabotulinumtoxinA for urinary incontinence from neurogenic detrusor overactivity. J Urol.

[41] Torrance G (1976). Social preferences for health states: an empirical evaluation of three measurement techniques. Socioecon Plann Sci.

[42] Montejo AL, Correas-Lauffer J, Maurino J, Villa G, Rebollo P, Diez T, Cordero L (2011). Estimation of a multiattribute utility function for the Spanish version of the TooL questionnaire. Value Health.

[43] The Market Research Society M: **MRS Code of Conduct.** 2010. https://www.mrs.org.uk/standards/code_of_conduct/.

[44] European Pharmaceutical Market Research Association E: **EphMRA Code of Conduct.** 2014. http://www.ephmra.org/Professional-Standards.

[45] The Association for Qualitative Research A: **Qualitative research recruitment guidelines.** 2002. http://www.aqr.org.uk/refsection/recruitment-bestpract.shtml.

[46] Stiggelbout AM, Eijkemans MJ, Kiebert GM, Kievit J, Leer JW, De Haes HJ (1996). The ‘utility’ of the visual analog scale in medical decision making and technology assessment. is it an alternative to the time trade-off?. Int J Technol Assess Health Care.

[47] Deyo RA, Diehr P, Patrick DL (1991). Reproducibility and responsiveness of health status measures. statistics and strategies for evaluation. Control Clin Trials.

[48] Linacre J (2012). Winsteps® Rasch measurement computer program.

[49] StataCorp (2007). Stata Statistical Software: Release 10.

[50] National Institute for Health and Clinical Excellence (NICE) (2013). Guide to the methods of technology appraisal. 2013. Process and methods guides.

[51] Young TA, Yang Y, Brazier JE, Tsuchiya A (2011). The use of rasch analysis in reducing a large condition-specific instrument for preference valuation: the case of moving from AQLQ to AQL-5D. Med Decis Making.

[52] Hatoum HT, Brazier JE, Akhras KS (2004). Comparison of the HUI3 with the SF-36 preference based SF-6D in a clinical trial setting. Value Health.

[53] Mulhern B, Meadows K: **The construct validity and responsiveness of the EQ-5D, SF-6D and diabetes health profile-18 in type 2 diabetes.** *Health Qual Life Outcomes* 2014, **12:**42. 10.1186/1477-7525-12-42 PMC4304018 24661350

[54] Brazier JE, Kolotkin RL, Crosby RD, Williams GR (2004). Estimating a preference-based single index for the Impact of Weight on Quality of Life-Lite (IWQOL-Lite) instrument from the SF-6D. Value Health.

[55] Bansback N, Tsuchiya A, Brazier J, Anis A: **Canadian valuation of EQ-5D health states: preliminary value set and considerations for future valuation studies.** *PLoS One* 2012, **7:**e31115. 10.1371/journal.pone.0031115 PMC3273479 22328929

[56] van Nooten FE, Koolman X, Brouwer WB (2009). The influence of subjective life expectancy on health state valuations using a 10 year TTO. Health Econ.

[57] Dolan P, Roberts J (2002). To what extent can we explain time trade-off values from other information about respondents?. Soc Sci Med.

