# Supplementary material for: Development of the Incontinence Utility Index: estimating population-based utilities associated with urinary problems from the Incontinence Quality of Life Questionnaire and Neurogenic Module
Source: Health Qual Life Outcomes. 2014 Oct 8;12:147. doi: 10.1186/s12955-014-0147-7 (PMC4196092; doi:10.1186/s12955-014-0147-7)
Supplement: Additional file 1 — Estimating a multi-attribute utility function (MAUF) for the abbreviated health state classification resulted from the I-QOL and its neurogenic module. [file 12955_2014_147_MOESM1_ESM.docx]

## Additional File 1.

## *Estimating a multi-attribute utility function (MAUF) for the abbreviated health state classification resulted from the I-QOL and its neurogenic module.*

The analytical approach undergone in this research involved a number of steps:

First, quality of data was tested to check the consistency of participants’ ratings when comparing values reported in corner states against directly comparable intermediate states (reversals). Small deviations (± 0.1 points) in these ratings were admitted due to practical limitations in the precision of the scale.

Second, respondents were grouped according to the health state they rated as least desirable: Group A if respondents preferred being dead to health state W and Group B if respondents considered being dead as the least desirable health state. Next, individual preference scores were aggregated into normalized 10% trimmed Person-Mean A and Person-Mean B values to reduce the influence of outliers. The resulting scores were reviewed to check if end-of-scale bias adjustment for mean values was required (states with a mean value score over 0.75) [11]. It should be noted that Group A and B had different scales given the worst scenario applicable in each case (Group A: worst scenario= 0 to Perfect/ Full health= 1 and Group B: dead = 0 to Perfect/ Full health = 1).

Third, once the normalized values were obtained, a variety of functional forms (regression utility scores, non-linear transformation techniques and spline functions) were explored to convert VAS values (v) into utilities (u), confirming that the traditional power function form proposed before [46] was also the best choice for both groups:

$u=1-(1-v)^{\alpha}$ Equation 3

In addition, alpha coefficients (α) were calculated for both groups fitting the following regression:

$\ln(1-u)=\ln(1-v)\alpha$ Equation 4

From the results of these natural log transformations, the adjustments (R-squared not corrected for the mean) were checked and VAS values (v) could be transformed into utility scores (u).

Fourth, before calculating the overall Person-mean utility scores, it was necessary to re-scale person-mean B scores from dead= = 0.00 / P= 1.00 to W= 0.00 / P= 1.00 scale, using a linear transformation. Overall Person mean scores were then calculated, taking into account the prevalence proportion in Person-Mean A and Person-Mean B groups (both in W=0.00 / P= 1.00 scale):

Weighted uj= (nA* Person-Mean A uj + nB * Person-Mean B uj -re-scaled-)/N

Positive Linear transformation was then applied to re-scale the weighted scores u into a Dead=0.00 / P= 1.00 scale (u’).

Fifth, utility scores were converted to disutility scores (1-utility) to estimate c_j`_s where j= 1,…,5 from equation 1. At this point, c parameter could be estimated by iteratively solving equation 2: $1+c=\prod_{j=1}^{5} \left(1+cc_{j}\right)$.

Furthermore, the relationship between attributes was studied by testing whether the additive model was supported by data:

= 1, then c= 0. Equation 5

Sixth, the fitted multi-attribute disutility function was transformed into a MAUF and the final algorithm for the IUI was presented:

$u=\left(1+p\right)\prod_{i=1}^{5} w_{i}-p$ Equation 6

where *w*_i_ reflects the relative weight for each attribute and is calculated as:

$w_{i}=1+cc_{i}\bar{u}_{i}$ Equation 7

and *p* is a constant:

$p=-1-\frac{1}{c}$ Equation 8.
